# A case of limbic encephalitis presenting as a paraneoplastic manifestation of limited stage small cell lung cancer: a case report

**DOI:** 10.1186/1752-1947-4-408

**Published:** 2010-12-17

**Authors:** Ahmed Fahim, Mohammad Butt, Damian V McGivern

**Affiliations:** 1 Department of Cardiovascular and Respiratory Studies, Castle Hill Hospital, Cottingham, UK; 2 Department of Oncology, Castle Hill Hospital, Cottingham, UK; 3 Respiratory Medicine, Castle Hill Hospital, Cottingham, UK

## Abstract

**Introduction:**

The differential diagnosis of altered mental status and behavioral change is very extensive. Paraneoplastic limbic encephalitis is a rare cause of cognitive impairment, which should be considered in the differential diagnosis.

**Case presentation:**

A 64-year-old British Caucasian woman presented to our hospital with a 12-week history of confusion and short-term memory loss. She was hyponatremic with a serum sodium level of 128mmol/L. Moreover, there was evidence of left hilar prominence on the chest radiograph. A thoracic computed tomography scan showed left hilar opacity with confluent lymphadenopathy. A percutaneous biopsy confirmed a diagnosis of small cell lung cancer. There was no radiological evidence of brain metastasis on the computed tomography scan. In view of continued cognitive impairment, which was felt to be disproportionate to hyponatremia, a magnetic resonance imaging scan of the brain was undertaken. It showed hyperintense signals from both hippocampi, highly suggestive of limbic encephalitis presenting as a paraneoplastic manifestation of small cell lung cancer. She had a significant radiological and clinical response following chemotherapy and radiotherapy.

**Conclusion:**

This case highlights the importance of considering paraneoplastic syndromes in patients with neurological symptoms in the context of lung malignancy. If initial investigations fail to reveal the cause of cognitive impairment in a patient with malignancy, magnetic resonance imaging may be invaluable in the diagnosis of limbic encephalitis. The clinical presentation, diagnostic techniques and management of paraneoplastic limbic encephalitis are discussed in this case report.

## Introduction

The differential diagnosis of cognitive impairment in a patient with lung malignancy is extensive. Paraneoplastic neurological syndromes, including limbic encephalitis, should be suspected as a cause of altered behavior and short-term memory loss, if the more common causes (brain metastasis, biochemical derangement, infection or drug related delirium) have been excluded. We report a case of paraneoplastic limbic encephalitis (PLE) associated with limited stage small cell lung cancer, which highlights the importance of considering this entity as a cause of cognitive dysfunction in a patient with lung carcinoma.

## Case presentation

A 64-year-old British Caucasian woman with a medical history of fibromyalgia, hypertension and asthma presented to our hospital with collapse and brief loss of consciousness. Our patient had no recollection of the event, and she did not have a history of witnessed seizures. According to her family, she had experienced progressively worsening short-term memory for the previous three months. She was a lifelong smoker with a 50-pack-year history. Her medications included citalopram, co-amilozide, salbutamol and beclomethasone inhalers.

**Figure 1:**
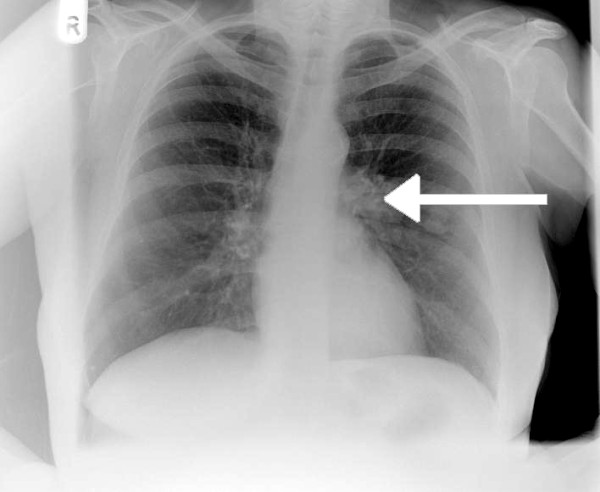
**Chest radiograph showing left hilar abnormality (arrow) and opacity in the left mid zone**.

**Figure 2:**
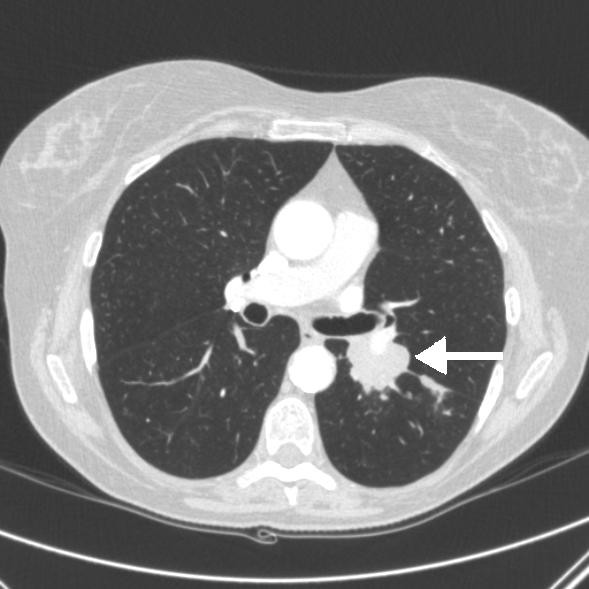
**Computed tomography (CT) scan showing parenchymal opacity in the apical segment of the left lower lobe (arrow), highly suggestive of lung malignancy**.

**Figure 3:**
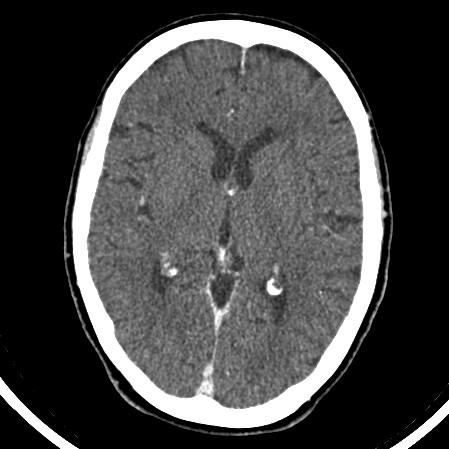
**Computed tomography (CT) image of our patient's brain on presentation, without significant acute pathology**.

**Figure 4:**
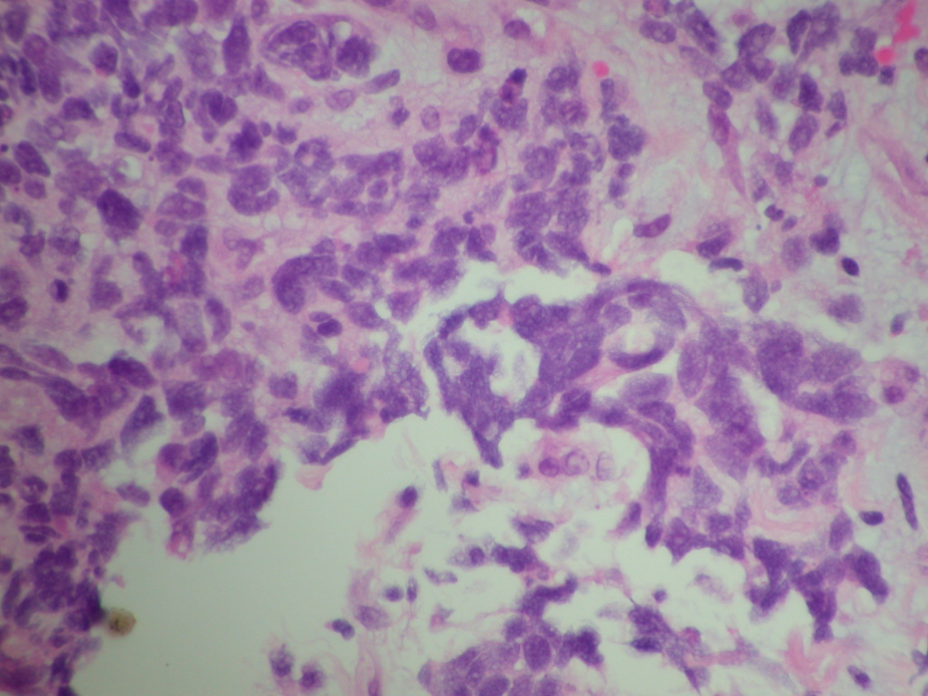
**Histology from a computed tomography (CT)-guided lung biopsy showing infiltration of the left lower lobe with neoplastic cells, consistent with small cell lung cancer**.

**Figure 5:**
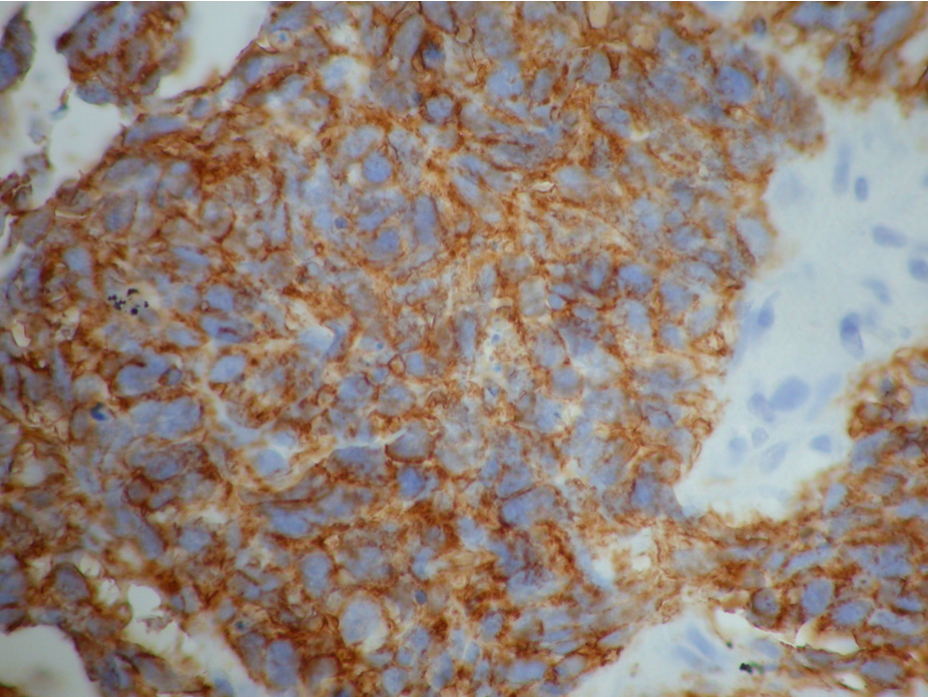
**Immunohistochemistry of a lung biopsy specimen showing positive staining with CD56, suggestive of small cell carcinoma of lung**.

**Figure 6:**
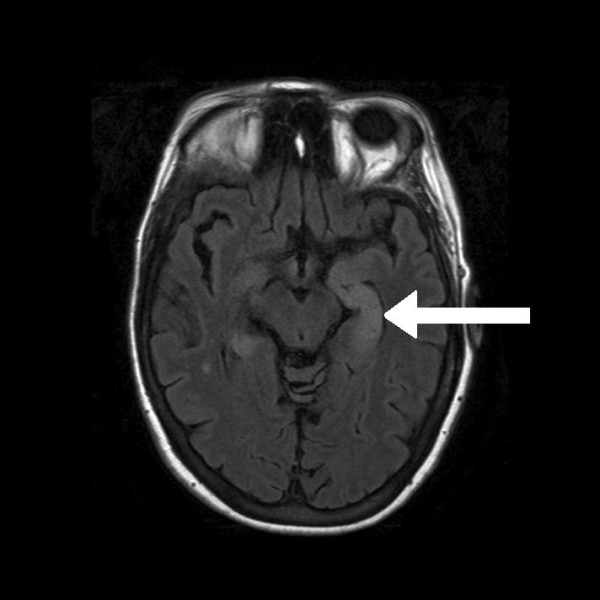
**Axial fluid attenuation inversion recovery (FLAIR) MRI of the brain showing a bright signal from the medial temporal lobe on the left side (arrow) consistent with limbic encephalitis**.

**Figure 7:**
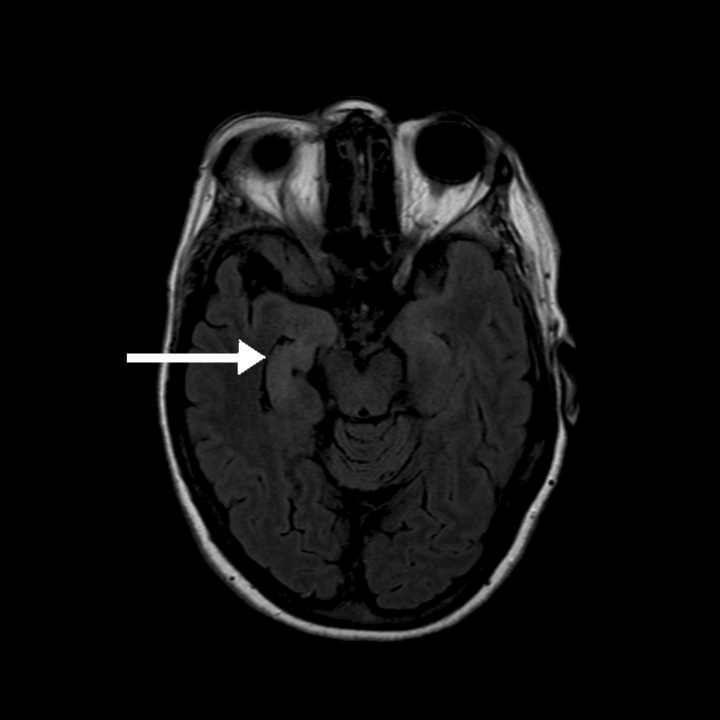
**Hyperintense signal from the hippocampus/medial temporal lobe on the right (arrow)**.

On examination, she was hemodynamically stable with pulse rate of 60 beats/minute and blood pressure of 107/75. Oxygen saturations were 95% on air. Her abbreviated mental test score was 7/10. On neurological examination there was no evidence of nystagmus, impaired coordination, sensory loss or muscle wasting. The rest of her systemic examination results were within normal limits. There was evidence of significant postural hypotension contributing to the clinical presentation of collapse and brief loss of consciousness. An electrocardiogram showed a normal sinus rhythm. However, a chest radiograph (Figure 1) was abnormal with left hilar shadowing. A biochemical profile showed hyponatremia with a serum sodium level of 128mmol/L. In view of her significant smoking history the most likely diagnosis was bronchogenic carcinoma with brain metastasis, and so a computed tomography (CT) scan of the thorax and head was arranged. The thoracic CT scan (Figure 2) revealed confluent left hilar lymphadenopathy encasing the left lower lobe pulmonary artery, and a parenchymal opacity in the left lower lobe was highly suggestive of bronchogenic carcinoma. The contrast-enhanced CT scan of her head (Figure 3) did not show any significant abnormality. Flexible fiber-optic bronchoscopy results were normal and bronchial washings were negative for malignant cells. We therefore performed a CT-guided biopsy (Figure 4) of the left lower lobe lesion. It was suggestive of neoplastic infiltration of lung parenchyma by small cell carcinoma. Furthermore, immunohistochemistry showed positive staining with CD56 (Figure 5), pancytokeratin, chromogranin and thyroid transcription factor 1 (TTF1), consistent with a diagnosis of small cell lung cancer. As there was no improvement in her cognitive function, a lumbar puncture was performed. The results of a cerebrospinal fluid (CSF) examination were unremarkable. In view of her persistent neurological symptoms, an MRI scan of the brain was performed that showed numerous small foci of increased T2-weighted signals scattered throughout the cerebral white matter, particularly in the frontal and parietal areas. Axial fluid attenuation inversion recovery (FLAIR) sequences showed hyperintense signals from the medial temporal lobe on the left (Figure 6) and right side (Figure 7), consistent with the radiological diagnosis of limbic encephalitis. There was no evidence of metastatic disease. A diagnosis of paraneoplastic limbic encephalitis associated with limited stage small cell lung cancer was made based on the clinical and radiological results. Our patient was treated with prophylactic cranial irradiation followed by platinum-based chemotherapy. Her cognitive function improved considerably over the course of next few months and her condition remained stable 18 months after presentation.

## Discussion

Paraneoplastic limbic encephalitis, first described as a clinical entity in 1968 [1] is characterized by short-term memory deficits, mood and behavioral changes and relative preservation of other cognitive functions. There may be seizures, which are most often partial complex in nature. Moreover, hypothalamic involvement can manifest with hyperthermia, hyperphagia or pituitary hormonal deficits [2]. Patients with PLE often present with symptoms of neurological involvement distant from the limbic system (commonly brainstem and cerebellum). Bakheit and colleagues [3] found that only 32% of patients had isolated limbic encephalitis. The neurological symptoms often precede identification of the tumor by weeks or months and the non-specific nature and diversity of symptoms add to the difficulty in diagnosing this rare clinical entity.

The most frequent neoplasms associated with PLE are small cell lung cancer, testicular tumors, thymoma, Hodgkin's lymphoma and breast cancer. In an analysis of 50 patients with PLE, Gultekin and colleagues [4] found that lung cancer was the most common neoplasm identified in 50% of cases, followed by testicular and breast carcinoma in 20% and 8%, respectively. The neurological symptoms preceded the diagnosis of cancer in approximately two-thirds of patients with a median duration of three and a half months.

Paraneoplastic limbic encephalitis can pose a diagnostic challenge in patients with cognitive impairment, especially when there is no evidence of malignant disease, and can be easily mistaken for viral encephalomyelitis or rapidly progressive neurodegenerative disease. Even in the presence of malignancy, the neurological symptoms can be easily attributed to cranial metastases.

An MRI scan of the brain is the most sensitive radiological investigation to diagnose limbic encephalitis. Typically, it shows hyperintense lesions in the medial temporal lobes and these are best visualized in T2 and axial FLAIR sequences without significant contrast enhancement. A CSF examination is seldom diagnostic of this condition, and the most common findings are consistent with inflammatory changes (pleocytosis, increased protein, oligoclonal bands and increased immunoglobulin content). Anti-neuronal antibodies are frequently found in the serum or CSF of patients with PLE, but the absence of these antibodies does not exclude the diagnosis. The most common is the anti-Hu antibody, which is present in about 50% of patients with small cell lung cancer presenting with limbic encephalitis and the anti-Ta antibody associated with testicular cancer [4]. Moreover, the presence of antibodies can be predictive for a good response to immunosuppressive therapy [5].

Electroencephalography can be a useful tool in order to support the diagnosis of limbic encephalitis, as it can demonstrate focal or generalized slowing and/or sharp wave epileptiform activity predominantly in the temporal regions [6]. The treatment should be directed at the associated malignancy, which frequently improves the neurological symptoms and is superior to immunomodulatory therapy.

## Conclusion

Our patient's case highlights the importance of considering PLE in the differential diagnosis of altered mental status in patients with lung malignancy, if there is no readily identifiable cause of cognitive impairment on initial investigations. Prompt diagnosis and early treatment of malignancy provides the best chance of clinical improvement in patients with this rare disorder.

## Consent

Written informed consent was obtained from the patient for publication of this case report and any accompanying images. A copy of the written consent is available for review by the Editor-in-Chief of this journal.

## Competing interests

The authors declare that they have no competing interests.

## Authors' contributions

AF, DVM and MB contributed to the writing of the manuscript. All authors read and approved the manuscript.
